# Phubbing in Adolescents: Spanish Validation of the Phubbing Scale (PS)

**DOI:** 10.3390/ijerph21091257

**Published:** 2024-09-22

**Authors:** Noelia Barbed-Castrejón, Fermín Navaridas-Nalda, Cristina Ábalos-Villanueva, Oliver Mason, Javier Ortuño-Sierra

**Affiliations:** 1 Educational Sciences Department, University of La Rioja, 26004 Logroño, Spain; fermin.navaridas@unirioja.es (F.N.-N.); cristina.abalos@alum.unirioja.es (C.Á.-V.); javier.ortuno@unirioja.es (J.O.-S.); 2 Psychology Department, Surrey University, Guildford GU2 7XH, UK; o.mason@surrey.ac.uk

**Keywords:** Phubbing Scale, adolescents, validation, psychometric properties, smartphone

## Abstract

The Phubbing Scale (PS) is an instrument used to measure the frequency and extent of the behavior of ignoring someone you are with and giving attention to your mobile phone instead. However, there is insufficient evidence about the psychometric adequacy of the Spanish version of the instrument. The main goal of this research was to analyze the psychometric properties of PS in a representative sample of Spanish adolescents and young adults. A total of 1351 participants comprised the sample (42.78% females, age range = 12–21). Students were selected from different levels of education such as secondary school, high school, vocational training, or university. A convenient sample was used. The reliability of the scores was calculated by means of McDonald’s Omega. The evidence of the internal structure of the questionnaire was analyzed by means of confirmatory factor analysis (CFA). The measurement invariance of the instrument by gender and educational level was also calculated. In addition, Pearson’s correlations between phubbing and other indicators of mental health were also calculated. The goodness-of-fit indices for the two-factor model were good. The McDonald’s Omega coefficient for the total score was 0.787. Measurement invariance both by gender and educational level was found. The phenomenon of phubbing was found to have statistically significant correlations with emotional well-being, other mental health indicators, and with Problematic Internet Use (PIU), with the sole exception of the hyperactivity subscale of the SDQ. This study provided validity evidence for the Spanish version of the Phubbing Scale (PS), suggesting that PS is a reliable tool for quantifying phubbing in Spanish adolescents.

## 1. Introduction

Smartphones have become one of the primary ways to communicate and to interact socially among adolescents, as well as the main way of accessing information regarding the world around them. The age of the current generation means that they are ‘digital natives’ [1], meaning that they are growing up in a world mediated by internet connections. Furthermore, adolescence is a critical period for establishing the foundations of good mental health, healthy social relationships, and well-being for adulthood. It is also a period of great vulnerability, where many psychological problems like problematic internet use (PIU) [2,3] can arise. Therefore, it is also a particularly sensitive period for the prevention of mental health difficulties [4,5].

The concept of ‘phubbing’ refers to the behavior of individuals who pay more attention to their smartphones than to those around them [3]. This form of probably inadvertent social snubbing has been investigated in recent years, primarily focusing on individuals exhibiting it, known as ‘phubbers’ [6,7,8], and on those who are phubbed [9]. Moreover, phubbing is considered by many an inappropriate behavior that affects social interactions [10], has negative consequences on interpersonal communications, and affects well-being [11]. Phubbing can occur at any place or time as most people have the device within their reach during meetings, conferences, at school, or in social gatherings with friends and family [12]. Consequently, phubbing indicates to others that the individual is not engaged or not interested in the social environment [13]. This phenomenon is relatively new to research [14], but there is a growing interest in its incidence, the way it occurs, and its consequences for others [15,16]. It is part of a wider context of smartphone use often associated with problematic and addictive behaviors related to internet use [7,17,18,19]. For instance, the study of Karadag [7], revealed that an excessive use of smartphones is often associated with problematic and addictive behaviors related to internet use, and the studies of Alonso and Romero found that this problem can begin in adolescence and increase with age [17]. Other researchers have linked addictive behaviors related to internet use with an excessive use of smartphones [18,19].

Multiple researchers have associated phubbing with various mental health issues [20], personal well-being problems [21], and fear of missing out (FoMO) [22]. Furthermore, several instruments have been developed to measure phubbing both in adolescent and adult populations, including the General Scale of Phubbing (GSP) and the General Scale of Being Phubbed (GSBP) [9]. The Phubbing Scale (PS) [7] is a widely used instrument with 10 items divided into two different dimensions: (1) Communication and (2) Obsession. Different studies have analyzed the psychometric adequacy of the PS across different populations [23,24,25,26,27,28,29,30,31]. For instance, Blachnio et al. [8] analyzed the internal structure of the PS across 20 countries and found a two-factor structure as the most adequate, confirming the original structure [7]. In addition, the work of Kim et al. [25] gathered evidence about the internal structure of the PS in a Korean population (PS-K), whereas Hwang et al. [24] validated the PS-K specifically for mothers. Blanca and Bendayan [23] analyzed the psychometric properties of the Spanish PS in adults, similarly finding a two-factor structure as the most satisfactory. In addition, they found that phubbing was associated with indicators of internet addiction and FoMO. Overall, the PS seems to have a two-factor structure, with correlated factors.

Measurement invariance (MI) across relevant variables, a critical aspect of an instrument, has also been studied [29,32]. For instance, García Castro et al. [32] found that the PS-8 was invariant across genders. Lin et al. [29] also found that the PS-8 was invariant across both gender and country. In this regard, Blachnio et al. [31] found MI in the PS-8, but only after eliminating three countries from the study.

As it can be seen, several studies have gathered evidence about the internal structure, the internal consistency of the scores, and the measurement invariance of the PS. However, knowledge about adolescent and young adult populations are still limited, with no reports of this in Spain.

Given the crucial importance of this period in the future emotional and mental well-being of adolescents, and the possible impact of phubbing on their correct social and psychological development [23,33], the main objective of this article was to analyze the psychometric properties of the Spanish version of the PS in a representative sample of adolescents. Therefore, the specific objectives were (a) to estimate descriptive statistics and rates of phubbing behaviors; (b) to analyze the internal structure of the PS; (c) to study the reliability of scores on the PS; (d) to gather evidence about the MI of the PS with attention to gender and educational level; and (e) to analyze the relationship between phubbing and other indicators of mental health, well-being, and socioemotional adjustment.

## 2. Materials and Methods

### 2.1. Participants

An initial sample of 1374 adolescents and young adults aged 12 to 21 took part in this research. Those participants with more than 3 missing values (*n* = 23) were deleted from the initial sample. Thus, the final sample was composed of 1351 participants. They were attending different levels of education such as secondary school, high school, vocational training, or university. The researchers chose these participants conveniently, meaning they selected them based on availability and accessibility. In terms of gender distribution, there were slightly more males (54.25%) than females (42.78%), with less than 1% identifying as ‘other’ and almost 2% opting not to specify their gender. About 20% of the participants were balancing their studies with part-time employment or internships, indicating that education was not their only or main activity.

### 2.2. Instruments

The Phubbing Scale (PS) [7] comprises 10 items rated on a Likert-type scale with five options (1 = Never; 5 = Always). The assessment of phubbing behavior was categorized into two factors: Communication Disturbance (CD) and Phone Obsession (PO) [8]. Although the studies have demonstrated satisfactory psychometric properties, they remain somehow limited in terms of diverse sample development [23]. Furthermore, there is not a specifically validated version designed for Spanish adolescent students. Blanca and Bendayan [23] previously adapted and validated the PS for a Spanish adult population, and their version has been used for this study, as it was considered adequate for adolescents and young people.

The Strengths and Difficulties Questionnaire (SDQ) [34] is a self-reported scale consisting of 25 items, distributed across five subscales, which contains five items each: emotional problems, behavior problems, peer problems, hyperactivity, and prosocial behavior. The translated and validated Spanish version of the scale has demonstrated satisfactory psychometric properties in adolescents [35].

The Rosenberg Self-Esteem Scale (RSE), developed by Rosenberg in 1965, is a widely used tool for assessing an individual’s self-esteem. Comprising 10 statements related to a person’s self-concept and self-evaluation, the respondents rate each statement on a four-point scale, ranging from “strongly agree” to “strongly disagree”. Generally, a higher score on the RSE indicates a heightened perception of self-esteem [36]. For this research, the Spanish version of the scale was used [37].

The Interpersonal Emotion Regulation Questionnaire (IERQ), developed by Hoffman et al. (2016), comprises 20 items categorized into four factors: Enhancing Positive Affect, Perspective Taking, Soothing, and Social Modeling. These factors are associated with the tendency to seek out others for amplifying feelings of happiness and joy, using interpersonal interactions as a reminder to avoid worries and find comfort, and learning from others how to manage specific situations, respectively [38]. In this research, the Spanish adaptation of the IERQ was conducted [39].

The Compulsive Internet Use Scale (CIUS) is a 14-item self-assessment scale designed to measure the severity of Internet addiction and/or compulsive, pathological, or another problematic Internet use (PIU). Each question uses a 5-point Likert scale, ranging from 0 (‘never’) to 4 (‘very often’), resulting in a total score that indicates severity of PIU [40].

The Personal Wellbeing Index-School Children (PWI-SC) is a self-administered scale created to assess subjective well-being and quality of life (QoL) in school-age children and adolescents. It comprises seven items gauging happiness in various life domains: standard of living, health, personal achievements, relationships, personal safety, community connection, and future security. Participants rate each item on a scale from 0 to 10, with 0 indicating “Very Sad” and 10 reflecting “Very Happy” [41]. The Spanish version of PWI-SC, used in this research, showed adequate psychometric properties in previous studies [42].

### 2.3. Procedure

The surveys were given to the students while they were at their educational institutions. A trained researcher gave instructions to complete the questionnaires, which were completed in about 30 min. Most of the participants (81%) used mobile devices to respond to the surveys, while the remaining participants used traditional paper questionnaires. Informed consent was obtained for those participants under 18 years old. The study was conducted with the approval of the Research Ethics Committee at the University of La Rioja (Code: inf_CE_46_2023) The database will be preserved by the PRISMA research team (a psychology research group based at the University of La Rioja) for the amount of time necessary to finish the entire research and at least the next 20 years in order to allow for database comparison.

### 2.4. Data Analysis

We studied the descriptive statistics and the percentage distribution of the PS items. Then, we analyzed the evidence of the internal structure of the questionnaire by means of confirmatory factor analysis (CFA). To this aim, we studied the one-dimensional model, the two-dimensional model [7,8], and a second-order factor model. Since the models did not show adequate goodness-of- fit indices, we also analyzed the two-dimensional model, allowing for correlated errors between items 3 and 5. We only allowed the correlated error between 2 items, so the model was far from being fully saturated. We used Muthén’s quasi-likelihood estimator [42]. We used the Chi square (χ^2^), Confirmatory Factor Index (CFI), Tucker–Lewis Index (TLI), Root Mean Square Error of Approximation (RMSEA), and Standardized Root Mean Square Residual (SRMR) as goodness-of-fit indices. Hu and Bentler [43] proposed that RMSEA values should be under 0.80 for a good model fit. In addition, CFI and TLI values over 0.95 or more are preferred, but values over 0.90 could be considered acceptable. For SRMR, values less than 0.08 should be accepted. Then, we analyzed the internal consistency of the scores. In addition, we tested the measurement invariance (MI) of the PS with successive multigroup CFAs. Delta parameterization was used [44]. To this aim, we conducted successive multigroup CFAs across gender and educational levels. As the ∆χ^2^ has shown several limitations due to the fact that it is sensitive to sample size, we followed the increase or decrease in CFI values (∆CFI), suggested by Cheung and Rensvold [45], to determine if nested models are practically equivalent. Once the internal structure was confirmed, we analyzed the internal consistency of the scores. We used McDonald’s Omega. Finally, we gathered information about the relationship of the PS with other variables by means of Pearson’s correlation. SPSS 17.0 (IBM Analytics, Armonk, NY, USA, 2016) and JASP Team (Amsterdam, The Netherlands, 2019) were used for data analyses.

## 3. Results

### 3.1. Descriptive Statistics for the PS and Prevalence Rates

The descriptive statistics of the PS and the percentages of different answers options are shown in Table 1.

### 3.2. Evidence of Validity Based on Internal Structure

We examined the goodness-of-fit indices for both the unidimensional and two-factor models. As can be seen in Table 2, the unidimensional model did not show an adequate fit. Although the two-factor model showed improved goodness-of-fit indices, they were still inadequate. We then analyzed a second-order factor solution and the two-factor model allowing for correlated errors between items 3 and 5. Both models displayed adequate goodness-of-fit-indices. We discarded the second order-factor as some factor loadings were not significant. Therefore, we retained the two-factor model with modifications as the most adequate solution.

We calculated factor loadings of the two-factor model with correlated errors. As shown in Table 3, all factor loadings were statistically significant and ranged from 0.374 (Item 5: “I think that I annoy my partner when I’m busy with my mobile phone”) to 0.82 (Item 1: “My eyes start wandering on my phone when I’m together with others”).

### 3.3. Measurement Invariance of the PS Scores by Gender and Age-Studies

Once the two-factor model with correlated errors was retained as the most satisfactory solution, we studied the MI of the PS scores, with attention to gender and age. With the aim to study the MI across educational level, we divided the sample into two different subgroups: non-university and university students. Then, we examined configural and strong MI. Differences in ΔCFI below 0.01 between the configural model and the strong model supported the hypothesis of strong MI across both gender and educational level (Table 2).

### 3.4. Study of the Reliability of the PS Scores

We studied the internal consistency of the PS scores by means of McDonald’s Omega coefficient. The Communication dimension showed a coefficient of 0.705, whereas Obsession revealed a coefficient of 0.709. The McDonald’s Omega for the total score was 0.787. In addition, we calculated discrimination indices that were all over 0.30. We also studied the internal consistency of the scores for the paper and computer-based forms. In the paper form, the scores were 0.72, 0.714, and 0.781, whereas in the computer-form, they were 0.711, 0.712, and 0.789, respectively, for the Communication, the Obsession, and the total score.

### 3.5. Relation of the PS Scores with Well-Being and Mental Health Variables

The study of the correlation between PS scores and different indicators of mental-health and PIU are shown in Table 4. As can be seen, the PS scores were statistically significant, and correlated with indicators of well-being and mental health, aside from the hyperactivity subscale of the SDQ. The PS dimensions and the PS total score were positively correlated with the total score of the SDQ, with the difficulty subscales of the SDQ, and with the total score of the CIUS; they were negatively associated with indicators of psychological well-being (total score of the PWI-SC) and self-esteem (total score of the Rosenberg).

## 4. Discussion

Phubbing behavior among adolescents and young adults is becoming a global issue [46]. This behavior impacts social interaction quality [10], personal well-being [47], and is associated with mental health issues [20]. Nonetheless, the research about this phenomenon is at an early stage, especially during the critical period of adolescence. Therefore, the present research aimed to analyze the psychometric properties of the PS as pertains to the current generation of digitally native young people.

The results of the present study indicate that phubbing is a highly prevalent behavior among adolescents and young adults. Prevalence rates were high for both dimensions of phubbing: Obsession and Communication. In addition, participants selected the options always or almost always in the majority of PS items. International studies have indicated that phubbing was a prevalent problem which needed further research [48,49,50], and our findings are similar. Providing evidence about this phenomenon can allow the establishment of a starting point to compare results of other research in different countries and populations and to better understand the growing phenomenon of phubbing and its implications for developing strategies to correct the consequences of a bad use of smartphones.

In terms of factorial structure, CFA confirmed a bi-dimensional structure, as shown by previous research in different countries performed by Blachnio et al. [8], consistent with the two dimensions defined by Karadag et al. [7]. The same structure was confirmed with Spanish adults [23]. Thus, the findings of this study provide valuable insights for utilizing the PS in school and university settings to investigate phubbing behaviors among these populations. It is worth noting that the two-factor model did not show adequate goodness and a model with the correlated errors of items 3 and 5 had to be considered. We also gathered evidence about the MI of the instrument. We found strong evidence of MI by gender and educational level, which contributes valuable information about the structural equivalence of the instrument across relevant variables. Studies about the MI of the PS are still limited [8,29,32]. Similarly to the results found in the present study, García Castro et al. [32] revealed that the PS was invariant across gender, although the PS-8 was used in their study. In addition, the PS-8 has been found to be invariant across countries, although no information is available about the measurement equivalent with regard to age or educational level. It is very useful to confirm MI by educational level, thus enabling comparison among, for instance, high school and university students.

With regard to the evidence of the relationship with other variables, the results indicated that PS scores were significantly associated with various indicators of well-being and mental health, including the SDQ difficulty subscales and the prosocial behavior subscale. The PS scores were also positively related with SDQ total score, and with the CIUS total score, which indicates that higher scores on phubbing were related with more psychological difficulties and with PIU. Moreover, a negative correlation was found with the total score of the PWI-SC and the total score of Rosenberg Scale, suggesting that psychological well-being, good self-esteem, and social adjustment could be protective factors for phubbing conduct. These results confirm previous findings about the positive correlation between phubbing and social interactions [10,13,15,16], mental health and personal well-being [20,21], and PIU and addictive conduct [17,19,51]. The study has several limitations including the use of self-report questionnaires and a single timepoint of measurement that present inherent problems to drawing causal conclusions. These kinds of instruments and designs limits the validity of the results. Therefore, future research could include objective measures (e.g., behavioral or neurobehavioral data). Also, longitudinal studies would allow the establishment of cause–effect relationships. This could extend our understanding of the phubbing phenomenon among adolescents. Finally, the results were obtained from a specific region using convenience sampling, so they should not be generalized to other cultural contexts. Future studies on the psychometric properties of the PS in different areas are necessary.

## 5. Conclusions

Despite these limitations, this study provides valuable insights into the increasingly prevalent phenomenon of phubbing [6,7,8] and its negative impact on psychological well-being [11]. This behavior impacts social interaction quality, personal well-being, and is associated with mental health issues. To date, evidence about instruments that allow to measure phubbing, with adequate psychometric properties, is still limited. The present work contributes valuable information about the evidence of the PS for its use in diverse educational levels in Spanish adolescent and young adult populations. The results of the present work can be taken forward to promote mental health. Early detection of behavioral and psychological issues, like phubbing, may allow the establishment of prevention strategies, specifically those related to mental health, social adjustment, and personal well-being as part of academic programs in educational contexts. Future research should further validate the psychometric properties of the PS across diverse regions and cultures, and explore its relationship with various mental health and well-being variables.

## Figures and Tables

**Table 1:** Prevalence and descriptive statistics of the Phubbing Scale (PS) for the total sample.

	Prevalence (%)						Descriptive Statistics			
Ítem	*n*	Never	Rarely	Sometimes	Almost Always	Always	Mean	SD	Skewness	Kurtosis
Communication										
1. My eyes start wandering on my phone when I’m together with others	1351	7.30	40.10	35.80	13.80	2.90	2.65	0.91	0.42	−0.03
2. I am busy with my mobile phone when I’m with my friends	1351	23.30	52.90	20.10	3.10	0.60	2.05	0.78	0.61	0.64
3. People complain about me dealing with my mobile phone	1351	48.93	33.16	11.62	4.52	1.78	1.77	0.95	1.3	1.38
4. I’m busy with my mobile phone when I’m with my family	1345	24.43	47.45	22.06	4.81	0.81	2.1	0.85	0.6	0.28
5. I think that I annoy my partner when I’m busy with my mobile phone (or family, if you do not have a partner)	1350	43.15	28.28	17.17	8.22	3.11	2	1.1	0.93	0.03
Obsession										
6. My phone is within my reach	1347	3.03	4.81	17.54	35.46	38.86	4.03	1.02	−1.03	0.68
7. When I wake up in the morning, I first check the messages on my phone	1341	17.91	19.91	19.62	21.91	20.65	3.07	1.4	−0.07	−1.28
8. I feel incomplete without my mobile phone	1341	31.90	33.68	20.36	10.07	4.00	2.21	1.12	0.72	−0.25
9. My mobile phone use increases day by day	1345	29.61	43.75	20.65	4.44	1.48	2.04	0.9	0.77	0.5
10. The time allocated to social, personal or professional activities decreases because of my mobile phone	1350	41.82	33.23	15.25	6.96	2.52	1.95	1.04	1.02	0.42

**Table 2:** Study of the internal structure and measurement invariance of the Phubbing Scale two-factor model across gender and educational level.

Model	χ^2^	df	CFI	TLI	RMSEA (CI 90%)	SRMR	ΔCFI
Baseline one-factor model	918.164	35	0.842	0.797	0.139 (0.132–0.147)	0.101	
Two-factor model	507.926	34	0.915	0.888	0.104 (0.096–0.112)	0.064	
Second-order factor	506.987	33	0.967	0.955	0.085 (0.077–0.093	0.066	
Two-factor model (correlated errors 3–5)	321.121	33	0.95	0.932	0.081 (0.073–0.089)	0.053	
Gender							
Male (*n* = 733)	252.466	33	0.962	0.957	0.080 (0.071–0.085)	0.052	
Female (*n* = 578)	272.351	33	0.958	0.953	0.081 (0.073–0.090)	0.057	
Configural invariance	327.156	66	0.955	0.953	0.078 (0.069–0.086)	0.054	
Strong invariance	401.067	102	0.948	0.954	0.067 (0.060–0.074)	0.057	−0.01
Educational Level							
Non-university (*n* = 755)	257.11	33	0.961	0.958	0.081 (0.072–0.086)	0.053	
University (*n* = 556)	264.68	33	0.951	0.908	0.082 (0.074–0.091)	0.056	
Configural invariance	329.604	66	0.953	0.952	0.081 (0.073–0.091)	0.055	
Strong invariance	400.658	102	0.949	0.950	0.080 (0.074–0.092)	0.056	−0.01

Note: χ^2^ = Chi square; df = degrees of freedom; CFI = Comparative Fit Index; TLI = Tucker–Lewis Index; RMSEA = Root Mean Square Error of Approximation; SRMR = Standardized Root Mean Square Residual; CI = Confidence Interval; ΔCFI = Change in Comparative Fit Index.

**Table 3:** Estimated saturation item parameters for the two-factor model with modifications.

Items	Estimate	Error	Lower	Upper
Communication				
1	0.832	0.017	0.800	0.865
2	0.739	0.016	0.703	0.771
3	0.485	0.017	0.516	0.519
4	0.673	0.016	0.641	0.704
5	0.374	0.018	0.421	0.408
Obsession				
6	0.544	0.017	0.510	0.577
7	0.621	0.016	0.589	0.653
8	0.719	0.016	0.687	0.755
9	0.739	0.017	0.706	0.771
10	0.571	0.017	0.538	0.603

**Table 4:** Correlation between the Phubbing Scale and different indicators of mental health and well-being.

	1	2	3	4	5	6	7	8	9	10	11	12
PS Total (1)												
PS Communication (2)	0.834 **											
PS Obsession (3)	0.885 **	0.480 **										
PWI-SC Total (4)	−0.123 **	−0.065 *	-0.142 **									
IERQ Total (5)	0.133 **	0.102 **	0.126 **	0.064 *								
Rosenberg Total (6)	−0.165 **	−0.127 **	−0.154 **	0.441 **	0.098 **							
CIUS Total (7)	0.504 **	0.410 **	0.455 **	−0.187 **	0.165 **	−0.256 **						
SDQ Emo (8)	0.177 **	0.125 **	0.179 **	−0.212 **	0.097 **	−0.369 **	0.226 **					
SDQ Cond (9)	0.135 **	0.182 **	0.056 *	−0.112 **	−0.01	−0.089 **	0.165 **	0.066 *				
SDQ Peer (10)	0.047	0.021	0.055 *	−0.142 **	−0.054	−0.174 **	0.133 **	0.145 **	0.103 **			
SDQ Hyper (11)	−0.022	−0.002	−0.037	−0.007	−0.012	−0.044	−0.014	0.138 **	0.098 **	0.038		
SDQ Pros (12)	0.041	0.097 **	−0.015	−0.05	−0.108 **	−0.017	0.087 **	−0.062 *	0.126 **	−0.082 **	−0.064 *	
SDQ Total (13)	0.171 **	0.160 **	0.134 **	−0.226 **	0.032	−0.330 **	0.248 **	0.696 **	0.530 **	0.515 **	0.510 **	−0.034

** The correlation is significant at 0.01 level (bilateral). * The correlation is significant at 0.05 level (bilateral). PS Total = total score of the Phubbing Scale, Ps Communication = Phubbing Scale Communication; PS Obsession = Phubbing Scale Obsession; PWI-SC Total = Personal Well-being Index-School Children; SDQ Emo = SDQ Emotional Problems; SDQ Cond = SDQ Conduct Problems; SDQ Peer = SDQ Peer Problems; SDQ Hyper = SDQ Hyperactivity; SDQ Pros = SDQ Prosocial.

## Data Availability

The data are not available as they contain sensitive information about human subjects and consent was not obtained for its dissemination.
